# Comparison of Effectiveness and Safety of Ustekinumab and Adalimumab As Induction or Maintenance Therapy in Patients With Moderate to Severe Crohn's Disease: A Systematic Review and Meta-Analysis

**DOI:** 10.7759/cureus.38277

**Published:** 2023-04-29

**Authors:** Venkata Sai Harshabhargav Chenna, Talwinder K Nagi, Zoilo K Suarez, Oscar L Hernandez, Maymona E Nageye, Nafisa Reyaz, Ibrahim Reyaz, Neelum Ali

**Affiliations:** 1 Medicine, University of Perpetual Help System Dalta, Las Pinas, PHL; 2 Internal Medicine, Florida Atlantic University Charles E. Schmidt College of Medicine, Boca Raton, USA; 3 Internal Medicine-Pediatrics, Avalon University School of Medicine, Willemstad, CUW; 4 Medicine, Jawaharlal Nehru Medical College and Hospital, Aligarh, IND; 5 Internal Medicine, Christian Medical College and Hospital Ludhiana, Ludhiana, IND; 6 Internal Medicine, University of Health Sciences, Lahore, PAK

**Keywords:** meta-analysis, clinical remission, crohn's disease, adalimumab, ustekinumab

## Abstract

This meta-analysis has been conducted to compare ustekinumab and adalimumab as induction or maintenance therapy in patients with moderate to severe Crohn's disease (CD). The current meta-analysis was conducted in accordance with the guidelines of the Preferred Reporting Items for Systematic Reviews and Meta-Analyses (PRISMA). Two investigators independently searched online databases including PubMed, Cumulated Index to Nursing and Allied Health Literature (CINAHL), and Cochrane Library for relevant articles published up to April 2023. The initial search terms were “ustekinumab,” “adalimumab,” and “Crohn’s disease". Three studies (with a total of 612 patients) were included in the present meta-analysis. We did not find any significant difference in clinical remission (OR: 1.31, 95% CI: 0.68-2.52), clinical response (OR: 1.39, 95% CI: 0.39-4.91), endoscopic remission (OR: 1.56, 95% CI: 0.66-3.64), and steroid-free remission (OR: 0.98, 95% CI: 0.67-1.42) between patients who received ustekinumab and patients who received adalimumab. In conclusion, this meta-analysis provides valuable insights into the efficacy and safety of ustekinumab and adalimumab in the treatment of moderate to severe CD. Our findings indicate that both drugs have similar effectiveness in achieving clinical remission, clinical response, radiological remission and steroid-free remission.

## Introduction and background

Crohn’s disease (CD), which is characterized by skip lesions and transmural inflammation, is a heterogeneous immune-mediated inflammatory condition predominantly affecting the intestine but capable of affecting any organ in the gastrointestinal tract from the mouth to the anus [1]. The annual incidence of CD can vary between 3 to 20 cases per 100,000 individuals [2], and the typical age for onset is around 30 years with two notable peaks: the first between 20-30 years and the second at 50 years [1]. The natural course of the disease follows a pattern of relapse and remission and can manifest in various ways, from inflammatory to stricturing and penetrating forms [3]. Continuous gut inflammation can harm the bowel and lead to changes in the disease's behavior, such as the development of strictures or fistulas, which can significantly impact patients' quality of life and level of disability [4]. The pathogenesis of CD is complex, involving environmental factors, genetic predisposition, microbial flora, and intestinal immune mechanisms [5]. The disease process is marked by increased production of pro-inflammatory cytokines [6], and thus the objective of treatment is to achieve quick recovery without using steroids and prevent future complications by suppressing the mechanisms causing inflammation. The choice of CD treatment depends on the disease's severity and type [7].

Currently, various biological therapies are available for treating CD, such as anti-tumour necrosis factor (TNF) agents (adalimumab and infliximab) or newer biological treatments, including ustekinumab. All of these therapies have been shown to be effective as maintenance and induction therapies when given as first- or second-line biological treatments in individuals with CD [8-9]. Since the introduction of TNF blockers, several other biological agents have been approved for treating CD, including ustekinumab, which is a humanized monoclonal antibody that targets interleukin (IL)-12 and IL-23, and is one of the latest agents to be approved for use in moderate to severe CD [10].

Although a network meta-analysis was conducted to compare the efficacy and safety of biologics in CD [11], indirect comparisons can be problematic because of differences in endpoints, populations, and inability to adjust for confounding variables. Therefore, a meta-analysis is required that includes studies with direct comparisons between adalimumab and ustekinumab in patients with CD. Secondly, since this meta-analysis was conducted, new studies have been performed with active comparisons between adalimumab and ustekinumab. Therefore, we are conducting this meta-analysis to pool available literature and compare ustekinumab and adalimumab as induction or maintenance therapy in patients with moderate to severe CD.

## Review

Methodology

The current meta-analysis was conducted in accordance with the guidelines of the Preferred Reporting Items for Systematic Reviews and Meta-Analyses (PRISMA).

Literature Search and Inclusion Criteria

Two investigators independently searched online databases including PubMed, Cumulated Index to Nursing and Allied Health Literature (CINAHL), and Cochrane Library for relevant articles published up to April 2023. The initial search terms were “ustekinumab,” “adalimumab,” and “Crohn’s disease” and medical subject headings (MeSH) terms were also used. No language restrictions were imposed. Additionally, the reference lists of all included studies were manually searched to identify other potentially eligible studies.

The following inclusion criteria were used: (a) study design: observational study or randomized-control trial (RCT); (b) study population: patients with Crohn’s disease; (c) intervention: ustekinumab (no matter what dose given); (d) comparison intervention: adalimumab and (d) outcome measures: clinical remission. We excluded studies conducted in individuals other than CD. We also excluded case reports, case series, editorials and review studies. Based on these criteria, two authors independently selected studies for further screening by reading abstracts or titles for all identified studies. Full-text text of eligible articles was retrieved and detailed assessment of articles was done using pre-defined inclusion and exclusion criteria.

Data Extraction

Two authors independently extracted the following data from each included study: first author name, publication year, country, study design, groups, sample size, and outcome measures. The primary outcome assessed in the present meta-analysis was clinical remission (as defined by the individual study). Other outcomes assessed included clinical response, steroid-free remission and endoscopic remission as defined by individual studies. Any disagreements between the two authors were resolved by consensus.

Data Analysis

Data analysis was conducted using RevMan software (version 5.4.1; Cochrane Collaboration, Oxford, UK). Outcomes were reported as odds ratios (ORs) with 95% confidence intervals. The statistical heterogeneity was assessed by conducting a chi-square test and I-squared statistics, where I2 values greater than 50% indicated significant heterogeneity among the study results. In case of significant heterogeneity, random-effects models were applied, and fixed-effect models were used when there was no significant heterogeneity. A p-value of less than 0.05 was considered statistically significant for determining the significance of the difference between ustekinumab and adalimumab.

Results

**Figure 1:**
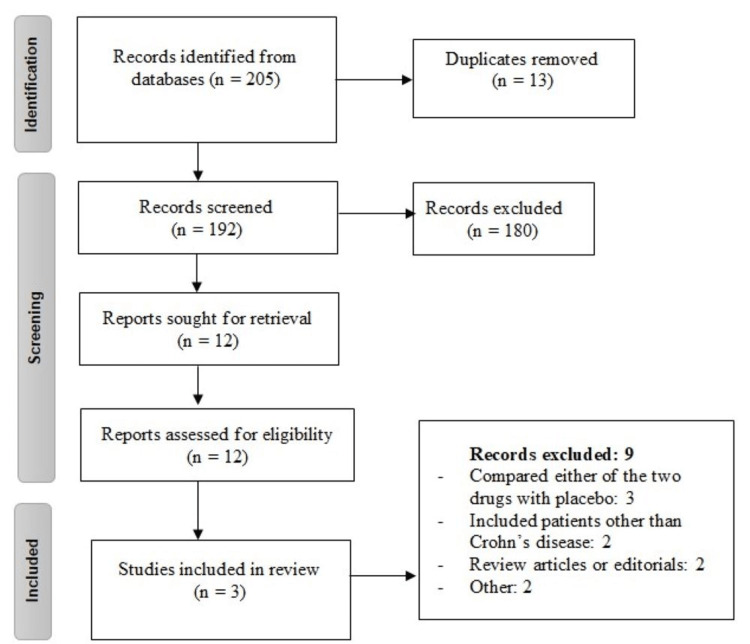
PRISMA flowchart of selection of studies PRISMA: Preferred Reporting Items for Systematic Reviews and Meta-Analyses

Two hundred and five articles were identified in the initial literature search, and 180 studies were excluded after title and abstract screening. The full text of 12 studies was obtained to perform a detailed assessment of the inclusion and exclusion criteria. Finally, three studies (with a total of 612 patients) were included in the present meta-analysis (Figure 1). The characteristics of the included studies are shown in Table 1.

**Table 1:** Characteristics of included studies RCT: randomized control trial

Author Name	Year	Study Design	Groups	Sample Size	Follow-up	Age (Years)	Males (%)
Ahmed et al [12]	2019	Prospective Cohort	Ustekinumab	65	16 Weeks	42.5 vs 40	34.8 vs 36.1
			Adalimumab	97			
Moens et al [13]	2022	Retrospective cohort	Ustekinumab	32	52 Weeks	45 vs 37	53 vs 56
			Adalimumab	32			
Sands et al [14]	2022	RCT	Ustekinumab	191	52 Weeks	37 vs 37.4	47 vs 49
			Adalimumab	195			

Meta-analysis of outcomes

Clinical Remission

**Figure 2:**
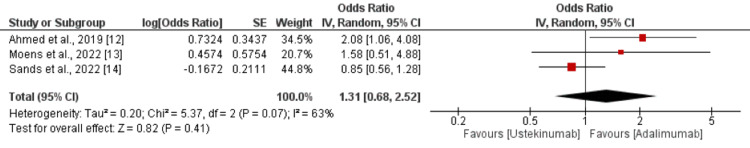
Forest plot comparing effects of ustekinumab and adalimumab on clinical remission Sources: References [12-14]

Three studies comprising 612 patients (of which 288 patients receive ustekinumab and 324 received adalimumab) were included in our analysis [12-14]. No significant difference was observed in odds of clinical remission between patients in the two groups (OR: 1.31, 95% CI: 0.68-2.52) as shown in Figure 2. High heterogeneity was reported among the study (I-square: 63%, p-value: 0.07).

Clinical Response

**Figure 3:**
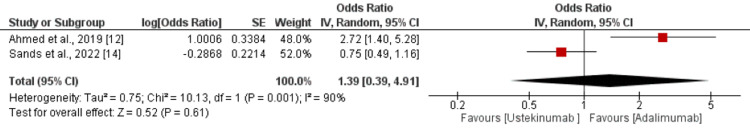
Forest plot comparing effects of ustekinumab and adalimumab on clinical response Sources: References [12,14]

Two studies assessed clinical response between the two groups and reported no significant difference between patients who received ustekinumab and adalimumab (OR: 1.39, 95% CI: 0.39-4.91) as shown in Figure 3. High heterogeneity was reported among the study results (I-square: 90%).

Steroid-Free Remission and Endoscopic Remission

**Figure 4:**
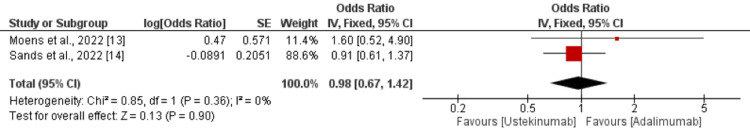
Forest plot comparing effects of ustekinumab and adalimumab on steroid-free remission Sources: References [13,14]

**Figure 5:**
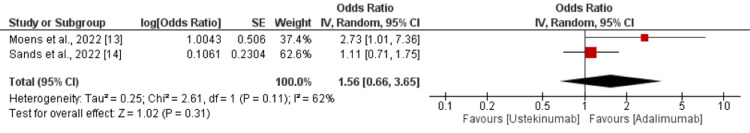
Forest plot comparing effects of ustekinumab and adalimumab on endoscopic remission Sources: References [13,14]

Two studies were included in the pooled analysis of comparing odds of steriod-free remission in patients receiving ustekinumab and adalimumab. No significant differences were found between the two study groups (OR: 0.98, 95% CI: 0.67-1.42) as reported in Figure 4. No heterogeneity was reported among the study results (I-square: 0%). No significant differences were found between the two study groups in terms of endoscopic remission (OR: 1.56, 95% CI: 0.66-3.64) as reported in Figure 5. High heterogeneity was reported among the study results (I-square: 62%). 

Safety Analysis

Moens et al. [13] reported no significant difference between the two groups in terms of adverse events (24 in each group) within the first year of treatment. No serious infections were reported nor were any death reported in any of the two groups. Sands et al. [14] reported 80% and 78% adverse events in ustekinumab and adalimumab groups respectively. Serious adverse events were reported in 16% of patients in the ustekinumab group and 13% of patients in the adalimumab group. No deaths were reported in any of the two groups during the one-year follow-up.

Discussion

This meta-analysis is the first to compare the efficacy and safety of ustekinumab and adalimumab in patients with moderate to severe CD. It included three studies with a pooled sample size of 612 patients. We did not find any significant difference in clinical remission, clinical response, radiological remission, and steroid-free remission between patients who received ustekinumab and patients who received adalimumab. Only one out of the three included studies showed that the odds of clinical remission were significantly higher in the adalimumab group compared to the ustekinumab group. This was because most patients treated with ustekinumab in this study were anti-TNF experienced, while patients who received adalimumab almost exclusively consisted of biological-naive patients. Thus, the differences in the population between the two groups may have contributed to these differences in findings compared to the other two studies.

The development of drugs that target TNF was a major step forward in treating CD, an immune-mediated inflammatory disease with multiple causes. However, despite the success of these drugs in bringing about significant clinical improvement and remission rates, roughly 30-40% of patients do not initially respond to them, and 20-30% of patients lose their response over time [15]. To address this group of patients, newer biological agents have become an important treatment option [16]. Ustekinumab is a fully human monoclonal antibody that works by blocking the IL-12/IL-23 p40 subunit and preventing it from interacting with the common receptor IL-12Rβ1, resulting in lower cytokine production and a decrease in the inflammatory process that characterizes the pathogenesis of CD [17].

Several studies have examined the efficacy of ustekinumab in patients with moderate to severe Crohn's disease who did not respond to traditional treatments or had adverse reactions to TNF inhibitors. These investigations revealed that ustekinumab was effective in enhancing clinical, laboratory, radiological, and endoscopic indicators of disease activity in patients with severe, unmanageable Crohn's disease that did not respond to TNF inhibitors [18,19]. However, there is a lack of studies comparing ustekinumab and adalimumab.

Overall, the safety profile was consistent across both groups, as none of the included studies reported a significant difference between the two groups in terms of adverse events and serious adverse events. Additionally, no deaths were reported in any of the patients in the two groups during the one-year follow-up. The most common adverse event reported by Sands et al [14] was infection in 34% and 41% of the ustekinumab and adalimumab groups respectively. Moens et al. [13] reported that in any eight events of infections in ustekinumab and 10 events of infections in adalimumab groups.

The present meta-analysis has certain limitations. Firstly, only three studies were included in the meta-analysis, and only one was an RCT. Secondly, certain outcomes, such as immunogenicity, were not assessed by the majority of the studies. Therefore, we were not able to assess this important outcome. Thirdly, due to limited data available, we were not able to perform a subgroup analysis, such as the effect of drugs in patients with early and delayed disease courses. Further studies are required to determine immunogenicity and treatment retention in both groups, as these two properties affect the long-term efficacy of drugs. The findings of the present meta-analysis support the use of effective and safe biologic agents for patients with moderate to severe active CD and reinforce the requirement for direct active-comparator studies for CD instead of indirect comparisons.

## Conclusions

In conclusion, this meta-analysis provides valuable insights into the efficacy and safety of ustekinumab and adalimumab in the treatment of moderate to severe CD. Our findings indicate that both drugs have similar effectiveness in achieving clinical remission, clinical response, radiological remission and steroid-free remission. Therefore, the choice of treatment between the two drugs may depend on other factors such as patient preference, cost, and previous treatment history. Additionally, given the limitations of the included studies, further research is warranted to explore the efficacy and safety of these drugs in different populations and over longer periods of follow-up. Overall, ustekinumab and adalimumab are both promising treatment options for patients with moderate to severe CD.
